# miR‐584‐5p regulates hepatocellular carcinoma cell migration and invasion through targeting KCNE2

**DOI:** 10.1002/mgg3.702

**Published:** 2019-05-01

**Authors:** Huamei Wei, Jianchu Wang, Zuoming Xu, Yuan Lu, Xianjian Wu, Chenyi Zhuo, Chuan Tan, Qianli Tang, Jian Pu

**Affiliations:** ^1^ Department of Pathology Affiliated Hospital of Youjiang Medical University for Nationalities Baise P. R. China; ^2^ Department of Hepatobiliary Surgery Affiliated Hospital of Youjiang Medical University for Nationalities Baise P. R. China; ^3^ Graduate College of Youjiang Medical University for Nationalities, Youjiang Medical University for Nationalities Baise P. R. China; ^4^ Youjiang Medical University for Nationalities Baise P. R. China

**Keywords:** cell behaviors, hepatocellular carcinoma, *KCNE2*, *miR‐584‐5p*, oncogenic miRNA

## Abstract

**Background:**

Hepatocellular carcinoma (HCC) is one of the most commonly diagnosed cancer type. This study was aimed to investigate the role of *microRNA‐584‐5p* (*miR‐584‐5p*) in regulating HCC progression.

**Methods:**

The expression of *miR‐584‐5p* in HCC cell lines was analyzed by quantitative real‐time polymerase chain reaction. Effects of *miR‐584‐5p* depletion on HCC cell proliferation, migration, and invasion in vitro were analyzed by cell counting kit‐8 assay, wound‐healing assay, and transwell invasion assay. *miR‐584‐5p* targeting potassium voltage‐gated channel subfamily E regulatory subunit 2 (*KCNE2*) was identified using bioinformatics algorithm and dual‐luciferase activity reporter assay. Kaplan–Meier Plotter website was used to investigate the effect of *miR‐584‐5p* or *KCNE2* expression on the overall survival of HCC patients.

**Results:**

In vitro functional assays showed *miR‐584‐5p* depletion decreased HCC cell proliferation, cell migration, and cell invasion. Moreover, *miR‐584‐5p* functions by directly targeting *KCNE2*, and it in turn, mediates the effects of *miR‐584‐5p* on HCC cell behaviors.

**Conclusions:**

These results demonstrated that *miR‐584‐5p* functions as an oncogenic miRNA in HCC.

## 1 INTRODUCTION

It is estimated that 841,000 new liver cancer cases occurred in 2018 worldwide (Bray et al., 2018). Hepatocellular carcinoma (HCC) represents 75%–85% of all liver cancer cases (Bray et al., 2018). China alone is estimated to account for approximately 50% of all HCC cases and therefore represents a heavy health challenge (Ashtari, Pourhoseingholi, Sharifian, & Zali, 2015). Five‐year overall survival for HCC patients is reported at the range of 30%–50% (Lee et al., 2006). Therefore, clarification of mechanisms underlying HCC tumorigenesis is an urgent need.

Cancer cell is characterized as uncontrolled cell growth, migration, and invasion that resulted from abnormal expression of many protein‐coding genes and abnormal activation signaling pathways (Agarwal, Narayan, Bhattacharyya, Saraswat, & Tomar, 2017; Jin et al., 2015). In addition to protein‐coding genes, noncoding RNAs including circular RNAs and microRNAs (miRNAs) are also reported to involve in HCC carcinogenesis (Xiong et al., 2018). It is estimated that miRNAs regulate >60% protein‐coding genes expression (Xiong et al., 2018). miRNAs are endogenous RNAs with the length of 18–24 nucleotides that can directly bind with the 3′‐untranslared region (3′‐UTR) of targeted gene and hence led to translation repression (Lin & Gregory, 2015). During HCC carcinogenesis, miRNAs were revealed to function as either tumor suppressor or promoter (Svoronos, Engelman, & Slack, 2016).

Recent years, abnormal expression of *microRNA‐584‐5p* (*miR‐584‐5p*) has been found in multiple human cancers including gastric cancer, neuroblastoma, medulloblastoma, and lung adenocarcinoma (Abdelfattah et al., 2018; Li et al., 2017; Xiang et al., 2015; Zhou et al., 2017). Importantly, *miR‐584‐5p* functions as oncogenic or tumor suppressive miRNA in a cancer type‐dependent manner (Abdelfattah et al., 2018; Li et al., 2017; Xiang et al., 2015; Zhou et al., 2017). For instance, *miR‐584‐5p* overexpression inhibits proliferation but promotes apoptosis in gastric cancer by targeting WW domain‐containing E3 ubiquitin protein ligase 1 (602307) (Li et al., 2017). In neuroblastoma, *miR‐584‐5p* suppressed cell growth, invasion, metastasis, and angiogenesis through recruiting enhancer of zeste homolog 2 (601573) to facilitate the methylation of matrix metalloproteinase 14 (600754) (Xiang et al., 2015). Restoration of *miR‐584‐5p* expression suppressed medulloblastoma cell growth, DNA damage, and caused cell cycle arrest by targeting eukaryotic translation initiation factor 4e family member 3 (609896) and histone deacetylase 1 (601241) (Abdelfattah et al., 2018). Another bioinformatic analysis study showed *miR‐584‐5p*, along with the other five miRNAs (*miR‐19b‐3p*,* miR‐21‐5p*,* miR‐221‐3p*,* miR‐409‐3p*, and* miR‐425‐5p*), was upregulated in lung adenocarcinoma, indicating the oncogenic role of *miR‐584‐5p* (Zhou et al., 2017). Yet, the biological function of *miR‐584‐5p* and the downstream target in HCC are still unclear.

In this work, we measured the expression of *miR‐584‐5p* in HCC cell lines and analyzed the effect of *miR‐584‐5p* expression on the overall survival of HCC patients. Furthermore, we conducted a series of in vitro studies to investigate the biological roles of *miR‐584‐5p* and potassium voltage‐gated channel subfamily E regulatory subunit 2 (KCNE2, 603796) in HCC. In addition, luciferase activity reporter assay and western blot assay were conducted to validate KCNE2 as a direct target of *miR‐584‐5p*.

## 2 MATERIALS AND METHODS

### 2.1 Cell culture

HCC cell lines (Hep3B, Bel‐7402, SK‐HEP‐1) and normal hepatocyte cell line LO2 bought from Cell Bank of the Chinese Academy of Sciences (Shanghai, China) were maintained in Dulbecco's Modified Eagle Medium (DMEM, Invitrogen, Thermo Fisher Scientific, Inc., Waltham, MA) supplemented with 10% fetal bovine serum (FBS, Invitrogen) in a 37°C humidified incubator containing 5% of CO_2_.

### 2.2 Cell transfection

miR‐584‐5p inhibitor (5′‐CUCAGUCCCAGGCAAACCAUAA‐3′) and corresponding negative control (miR‐NC, 5′‐ACAUCGAAGCCAAUUCAACCGC‐3′) were bought from GenePharma (Shanghai, China). Small interfering RNA targeting KCNE2 (si‐KCNE2, 5′‐CACCGGATTGGCAGCAGAAGTATAGCGAACTATACTTCTGCTGCCAATCC‐3′) and the corresponding negative control (siR‐NC, 5′‐AGTTACTCACCATCGGACGTCAACAACGGTTGCCAACGCGAATGTTGCTA‐3′) were also bought from GenePharma. Cell transfection was conducted using Lipofectamine 2000 according to the manufacturer's instructions. Cells were subjected to further assays after transfection for 48 hr.

### 2.3 Cell proliferation assay

Cell proliferation was analyzed by cell counting kit‐8 (CCK‐8) assay. Cells at the density of 5 × 10^3^ cells per well were seeded in 96‐well plate and incubated for 0, 24, 48, and 72 hr after seeding. CCK‐8 reagent (Beyotime, Haimen, Jiangsu, China) was added to the plate at the abovementioned time points and further incubation for 4 hr. Finally, we measured the optical density at 450 nm using a Microplate reader (Thermo Fisher Scientific, Inc.). Experiments were repeated in triplicates.

### 2.4 Wound‐healing assay

Cell migration was analyzed by wound‐healing assay. Cells were seeded to 6‐well plate and incubated until approximately 90% confluence. A plastic tip was used to create a wound at cell surface. Then, phosphate‐buffered solution buffer was used to remove cell debris. At 0 and 24 hr after wound creation, cell images were captured under an inverted microscope. Experiments were repeated in triplicates.

### 2.5 Transwell invasion assay

Cell invasion was analyzed by Transwell invasion assay. Cells in DMEM were seeded in the upper chamber coated with Matrigel (Corning, NY), while the lower chamber was filled with DMEM added with 10% FBS. After incubation for 24 hr, invasive cells at the lower chamber was fixed with 4% paraformaldehyde and stained with 1% crystal violet. Invasive cell numbers from five dependent fields were calculated under microscope. Experiments were repeated in triplicates.

### 2.6 Bioinformatic analysis and dual‐luciferase activity reporter assay

Targets of *miR‐584‐5p* was predicted by TargetScan. Among all these predicted targets, *KCNE2* was selected for further investigation. The wild‐type or mutant 3′‐UTR of *KCNE2* was cloned into a luciferase activity named pGL3 (Promega, Madison, WI). These vectors were designated as wt‐KCNE2 or mt‐KCNE2, respectively. Cells were then c‐transfected with wt‐KCNE2 or mt‐KCNE2 and miR‐584‐5p inhibitor or miR‐NC using Lipofectamine 2000. Relative luciferase activity was measured with dual‐luciferase activity reporter system (Promega) after transfection for 48 hr.

### 2.7 RNA extraction and quantitative real‐time polymerase chain reaction

Total RNA from cultured cells was isolated using Trizol reagent (Invitrogen). Then, these RNA sample was reverse transcribed into cDNA with PrimeScrip RT kit (Takara, Dalian, China). *miR‐584‐5p* expression level was quantified by TaqMan miRNA assays (Applied Biosystems, Foster City, CA). SYBR Green PCR Master Mix (Takara) was used to detect the expression level of *miR‐584‐5p* at an ABI 7500 system (Applied Biosystems, Foster City, CA). Relative expression level of *miR‐584‐5p* was normalized to U6 small nuclear RNA (*U6 snRNA*) and measured using 2^‐ΔΔCt ^method. The following thermocycling conditions were used: 10 min at 95°C; 40 cycles of 10 s at 95°C; 20 s at 63°C. Primers used in this work were as follows: *miR‐584‐5p* forward, 5′‐TTATGGTTTGCCTGGGACTGAG‐3′; reverse, 5′‐GCGAGCACAGAATTAATACGAC‐3′; *U6 snRNA* forward, 5′‐CTCGCTTCGGCAGCACA‐3′ and reverse, 5′‐AACGCTTCACGAATTTGCGT‐3′. Experiments were repeated in triplicates.

### 2.8 Protein extraction and western blot

Cultured cells were lysed with RIPA lysis buffer (Beyotime) according to the supplier's instructions to extract total proteins. Protein concentration was quantified with bicinchoninic acid Protein Assay kit (Beyotime). Equal amount of protein sample was separated using 10% sodium dodecylsulphate polyacrylamide gel electrophoresis and transferred to polyvinylidene difluoride membranes (Beyotime). Membranes were incubated at 4°C for overnight with corresponding primary antibodies (anti‐KCNE2: ab69376; anti‐GAPDH: ab181602; Abcam, Cambridge, MA). Then, membranes were incubated with horseradish peroxidase‐conjugated secondary antibodies (ab6721, Abcam) at room temperature for 2 hr. Bands were visualized using BeyoECL kit (Beyotime) and analyzed with Image J 1.42 software (NIH, Bethesda, MD). Experiments were repeated in triplicates.

### 2.9 KM Plotter analyze the effect of *miR‐584‐5p* and *KCNE2* expression on overall survival

Kaplan–Meier plotter (www.kmplot.com) was used to assess the effects of *miR‐584‐5p* or *KCNE2* expression on overall survival of HCC patients (Nagy, Lánczky, Menyhárt, & Győrffy, 2018). Cutoff value was auto‐selected in the algorithm. Log‐rank test was used to analyze difference in high or low *miR‐584‐5p* or *KCNE2* group.

### 2.10 Statistical analysis

Data were presented as mean ± standard deviation after analyzed at GraphPad Prism 6.0 (GraphPad Inc., San Diego, CA). Student's *t* test (two groups) and one‐way analysis of variance and Tukey post‐hoc test (multiple groups) were conducted to analyze difference in groups. Differences were defined as statistically significant when *p* < 0.05.

## 3 RESULTS

### 3.1 *miR‐584‐5p* expression was upregulated in HCC cell lines

**Figure 1:**
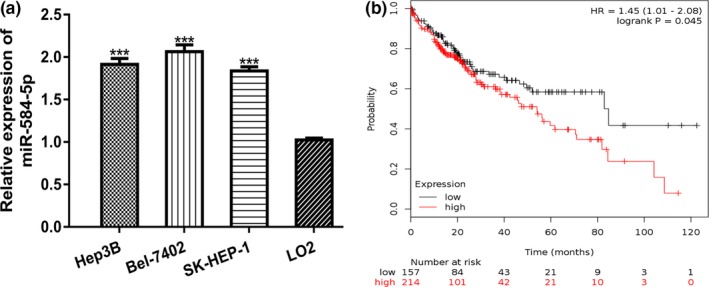
High expression of *miR‐584‐5p* in HCC. (a) *miR‐584‐5p* expression in HCC cell lines (Hep3B, Bel‐7402, SK‐HEP‐1) and normal hepatocyte cell line LO2 was analyzed by qRT‐PCR. (b) High *miR‐584‐5p* expression was correlated with overall survival of HCC patients. *miR‐584‐5p*, *microRNA‐584‐5p*; HCC, hepatocellular carcinoma; qRT‐PCR, quantitative real‐time polymerase chain reaction

We found *miR‐584‐5p* expression was significantly upregulated in HCC cell lines compared with the L02 cell line (Figure 1a). Moreover, high *miR‐584‐5p* expression was found correlated with poor overall survival of HCC patients (Figure 1b).

### 3.2 *KCNE2* expression was downregulated in HCC cell lines

**Figure 2:**
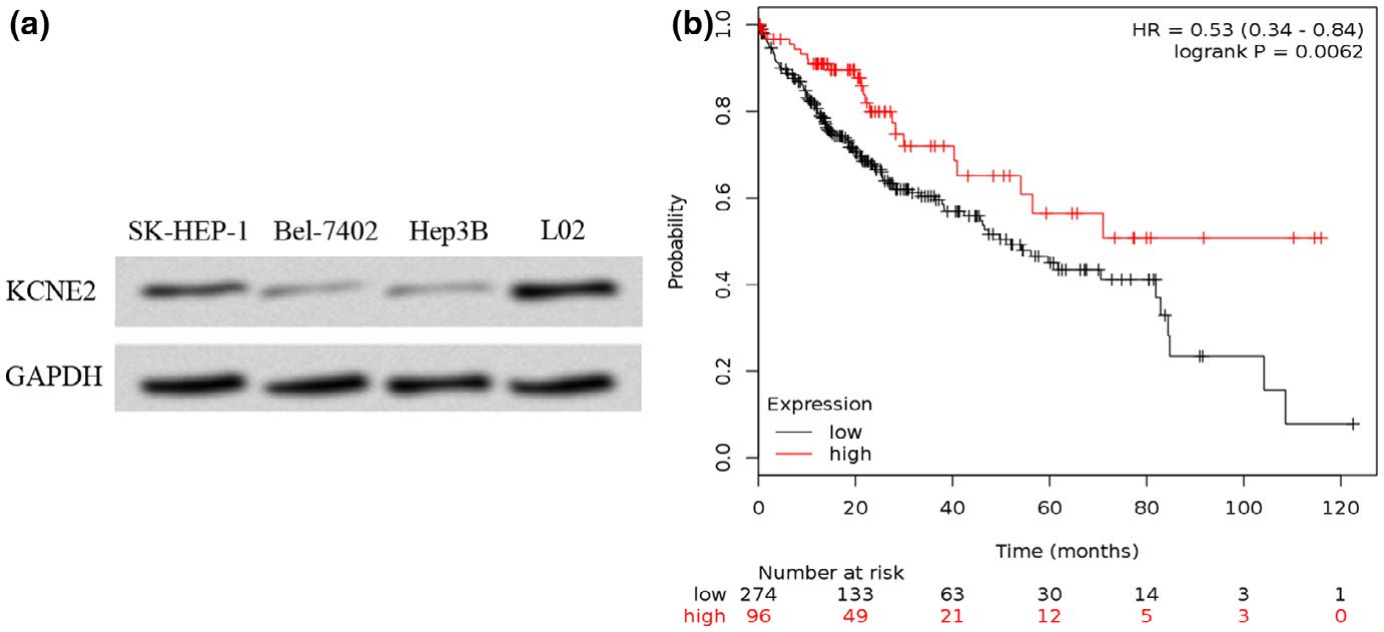
Low expression of *KCNE2* in HCC. (a) *KCNE2* expression in HCC cell lines (Hep3B, Bel‐7402, SK‐HEP‐1) and normal hepatocyte cell line LO2 was analyzed by western blot. (b) Low *KCNE2* expression was correlated with overall survival of HCC patients. KCNE2, potassium voltage‐gated channel subfamily E regulatory subunit 2; HCC, hepatocellular carcinoma

Then, *KCNE2* expression in HCC cell lines was examined by western blot. We showed *KCNE2* expression was downregulated in HCC cell lines compared with the L02 cell line (Figure 2a). In addition, we showed low *KCNE2* expression was a predictor for poor overall survival of HCC patients (Figure 2b).

### 3.3 KCNE2 was a direct target of miR‐584‐5p

**Figure 3:**
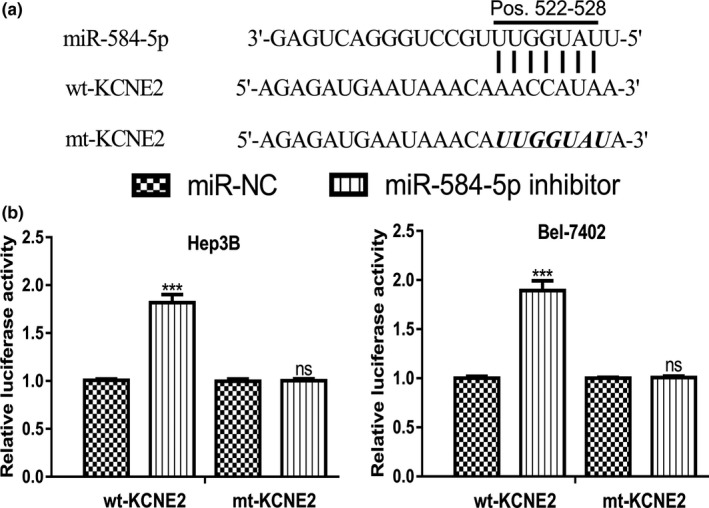
KCNE2 was a direct target of *miR‐584‐5p*. (a) Binding site between *miR‐584‐5p* and the 3′‐UTR of *KCNE2*. (b) Luciferase activity in cells transfected with miR‐584‐5p inhibitor or miR‐NC and wt‐KCNE2 or mt‐KCNE2. *miR‐584‐5p*, *microRNA‐584‐5p*; KCNE2, potassium voltage‐gated channel subfamily E regulatory subunit 2; UTR, untranslated region; wt, wild‐type; mt, mutant; miR‐NC, negative control miRNA

Since *miR‐584‐5p* expression was upregulated and *KCNE2* expression was downregulated in HCC, therefore we are interested to investigate whether KCNE2 was a direct target of *miR‐584‐5p*. Bioinformatic analyses showed 3′‐UTR of *KCNE2* contains a binding site for *miR‐584‐5p* (Figure 3a). Luciferase activity reporter assay revealed that the introduction of *miR‐584‐5p* inhibitor could increase the luciferase activity of cells transfected with wt‐KCNE2 (Figure 3b). However, it did not change the luciferase activity of cells transfected with mt‐KCNE2 transfection (Figure 3b).

### 3.4 *miR‐584‐5p* regulates HCC cell proliferation, migration, and invasion through targeting KCNE2

**Figure 4:**
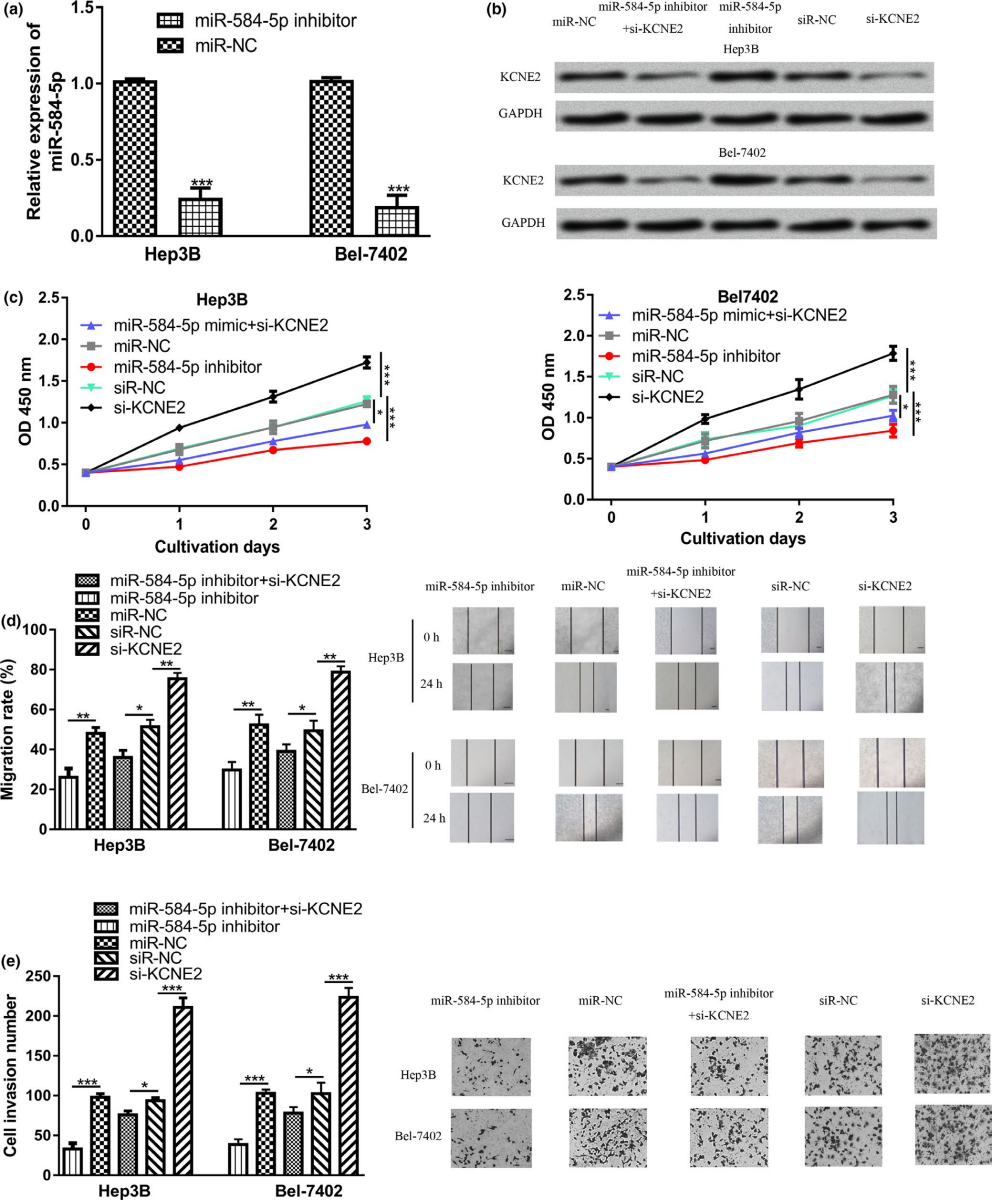
*miR‐584‐5p* regulated HCC cell proliferation, migration, and invasion through targeting KCNE2. (a) *miR‐584‐5p* expression in HCC cells transfected with miR‐584‐5p inhibitor or miR‐NC. (b) *KCNE2* expression. (c) Cell proliferation. (d) Cell migration. (e) Cell invasion in HCC cells transfected with miR‐584‐5p inhibitor, miR‐NC, si‐KCNE2, siR‐NC, or miR‐584‐5p inhibitor and si‐KCNE2. HCC, hepatocellular carcinoma; *miR‐584‐5p*, *microRNA‐584‐5p*; KCNE2, potassium voltage‐gated channel subfamily E regulatory subunit 2; miR‐NC, negative control miRNA; si‐KCNE2, small interfering RNA targeting KCNE2; siR‐NC, negative control siRNA

To test the role of *miR‐584‐5p* and KCNE2 on HCC cell behaviors, *miR‐584‐5p* inhibitor or si‐KCNE2 was used to manipulate the levels of miR‐584‐5p or KCNE2. It was found miR‐584‐5p inhibitor transfection decreased levels of *miR‐584‐5p* in HCC cell lines (Figure 4a). Meanwhile, *KCNE2* expression was upregulated by miR‐584‐5p inhibitor, while downregulated by si‐KCNE2 (Figure 4b). CCK‐8 assay showed that cell proliferation was inhibited by miR‐584‐5p inhibitor but promoted by si‐KCNE2 (Figure 4c). Wound‐healing assay showed that cell migration ability was decreased by miR‐584‐5p inhibitor but increased by si‐KCNE2 (Figure 4d). In addition, the introduction of miR‐584‐5p inhibitor decreased cell invasion ability (Figure 4e). The introduction of si‐KCNE2 increased HCC cell invasion ability (Figure 4e). Importantly, the si‐KCNE2 introduction could partially reverse the inhibitory effects of miR‐584‐5p inhibitor on HCC cell behaviors (Figure 4c‐4e).

## 4 DISCUSSION

Sustained cell growth is believed to be one of the key hallmarks of cancer that is characterized as imbalance of growth promotion and limitation (Hanahan & Weinberg, 2011). During this process, multiple molecules or signaling pathways were revealed to be abnormally activated (Hanahan & Weinberg, 2011). miRNA is reported to play crucial roles in the progression of human cancer including HCC (Chen et al., 2018; Sun et al., 2018; Yang et al., 2018). For instance, *miR‐133a* was found to be a tumor suppressive miRNA to inhibit HCC progression through targeting Fos‐related antigen 2 (601575)/transforming growth factor‐β/SMAD family member 3 (603109) signaling pathway (Sun et al., 2018). *miR‐302c‐3p* also exhibited a tumor suppressive role and inhibit HCC cell migration and invasion via targeting tumor necrosis factor receptor associated factor 4 (602464) (Yang et al., 2018). Moreover, *miR‐590‐5p* was found as the upstream regulator for yes‐associated protein 1 (606608) and directly regulate the chemo‐sensitive of HCC cells to transarterial chemoembolization (Chen et al., 2018). These studies outlined the importance of miRNAs in the carcinogenesis of HCC.

In this work, we showed that *miR‐584‐5p* expression was upregulated in HCC cell lines compared with normal cell line. We also showed knockingdown the expression of *miR‐584‐5p* could inhibit HCC cell proliferation, migration, and invasion in vitro. More importantly, we showed high *miR‐584‐5p* could result in poorer overall survival for HCC patients. These results indicated the oncogenic role of *miR‐584‐5p* in HCC, which is in consistent with its role in gastric cancer (Zhou et al., 2017).

To understand the role of miRNA, the identification of its downstream targets is essential (Chen et al., 2018; Sun et al., 2018; Yang et al., 2018). Multiple targets for *miR‐584‐5p* have been identified and validated in previous studies (Abdelfattah et al., 2018; Li et al., 2017; Xiang et al., 2015). Here, by bioinformatic analyses, we found KCNE2 was a potential target of miR‐584‐5p. The luciferase activity reporter assay confirmed this prediction. KCNE2 was previously identified to be downregulated in gastric cancer and the force expression of *KCNE2* could inhibit cancer cell proliferation and cell cycle progression (Abbott & Roepke, 2016; Yanglin et al., 2007). We found *KCNE2* expression was also downregulated in HCC cell lines compared with normal cell line in this work. Moreover, we showed low *KCNE2* expression was correlated with poor overall survival of HCC patients. In addition, knockdown the expression of *KCNE2* was able to promote HCC cell proliferation, migration, and invasion. More importantly, we showed the role of miR‐584‐5p on HCC cell behaviors was exerted through targeting KCNE2. The limitation of this study is that we did not validate our conclusion in human cohort. Therefore, we will try to recruit HCC patients into our study to investigate the expression and connection of *miR‐584‐5p* and KCNE2 in human tissues. Moreover, we will validate the oncogenic role of *miR‐584‐5p* in animal model and test whether targeting miR‐584‐5p is able to slow the growth of HCC.

In conclusions, our results indicated that *miR‐584‐5p* promotes HCC growth, migration, and invasion through targeting the expression of *KCNE2*. Our results provided novel insights into the mechanisms underlying the carcinogenesis of HCC and may provide novel therapeutic targets for HCC treatment.

## CONFLICT OF INTEREST

The authors declare that they have no conflict of interest.

## References

[mgg3702-bib-0001] Abbott, G. W. , & Roepke, T. K. (2016). KCNE2 and gastric cancer: Bench to bedside. Oncotarget, 7, 17286–17287. 10.18632/oncotarget.7921 26956055 PMC4951210

[mgg3702-bib-0002] Abdelfattah, N. , Rajamanickam, S. , Panneerdoss, S. , Timilsina, S. , Yadav, P. , Onyeagucha, B. C. , … Rao, M. K. (2018). MiR‐584‐5p potentiates vincristine and radiation response by inducing spindle defects and DNA damage in medulloblastoma. Nature Communications, 9(1), 4541 10.1038/s41467-018-06808-8 PMC6208371 30382096

[mgg3702-bib-0003] Agarwal, R. , Narayan, J. , Bhattacharyya, A. , Saraswat, M. , & Tomar, A. K. (2017). Gene expression profiling, pathway analysis and subtype classification reveal molecular heterogeneity in hepatocellular carcinoma and suggest subtype specific therapeutic targets. Cancer Genetics, 216–217, 37–51. 10.1016/j.cancergen.2017.06.002 29025594

[mgg3702-bib-0004] Ashtari, S. , Pourhoseingholi, M. A. , Sharifian, A. , & Zali, M. R. (2015). Hepatocellular carcinoma in Asia: Prevention strategy and planning. World Journal of Hepatology, 7(12), 1708–1717. 10.4254/wjh.v7.i12.1708 26140091 PMC4483553

[mgg3702-bib-0005] Bray, F. , Ferlay, J. , Soerjomataram, I. , Siegel, R. L. , Torre, L. A. , & Jemal, A. (2018). Global Cancer Statistics 2018: GLOBOCAN estimates of incidence and mortality worldwide for 36 cancers in 185 countries. CA: A Cancer Journal of Clinicians, 68(6), 394–424. 10.3322/caac.21492 30207593

[mgg3702-bib-0006] Chen, M. , Wu, L. , Tu, J. , Zhao, Z. , Fan, X. , Mao, J. , … Ji, J. (2018). miR‐590‐5p suppresses hepatocellular carcinoma chemoresistance by targeting YAP1 expression. EBioMedicine, 35, 142–154. 10.1016/j.ebiom.2018.08.010 30111512 PMC6154877

[mgg3702-bib-0007] Hanahan, D. , & Weinberg, R. A. (2011). Hallmarks of cancer: The next generation. Cell, 144(5), 646–674. 10.1016/j.cell.2011.02.013 21376230

[mgg3702-bib-0008] Jin, B. , Wang, W. , Du, G. , Huang, G. Z. , Han, L. T. , Tang, Z. Y. , … Zhang, S. Z. (2015). Identifying hub genes and dysregulated pathways in hepatocellular carcinoma. European Review for Medical and Pharmacological Sciences, 19(4), 592–601. 25753876

[mgg3702-bib-0009] Lee, J. G. , Kang, C. M. , Park, J. S. , Kim, K. S. , Yoon, D. S. , Choi, J. S. , … Kim, B. R. (2006). The actual five‐year survival rate of hepatocellular carcinoma patients after curative resection. Yonsei Medical Journal, 47(1), 105–112. 10.3349/ymj.2006.47.1.105 16502491 PMC2687566

[mgg3702-bib-0010] Li, Q. , Li, Z. , Wei, S. , Wang, W. , Chen, Z. , Zhang, L. , … Xu, Z. (2017). Overexpression of miR‐584‐5p inhibits proliferation and induces apoptosis by targeting WW domain‐containing E3 ubiquitin protein ligase 1 in gastric cancer. Journal of Experimental & Clinical Cancer Research, 36(1), 59 10.1186/s13046-017-0532-2 28431583 PMC5401563

[mgg3702-bib-0011] Lin, S. , & Gregory, R. I. (2015). MicroRNA biogenesis pathways in cancer. Nature Reviews Cancer, 15(6), 321–333. 10.1038/nrc3932 25998712 PMC4859809

[mgg3702-bib-0012] Nagy, Á. , Lánczky, A. , Menyhárt, O. , & Győrffy, B. (2018). Validation of miRNA prognostic power in hepatocellular carcinoma using expression data of independent datasets. Scientific Reports, 8(1), 9277. 29907753 10.1038/s41598-018-27521-y PMC6003936

[mgg3702-bib-0013] Sun, L. U. , Guo, Z. , Sun, J. , Li, J. , Dong, Z. , Zhang, Y. , … Yu, Z. (2018). MiR‐133a acts as an anti‐oncogene in Hepatocellular carcinoma by inhibiting FOSL2 through TGF‐β/Smad3 signaling pathway. Biomedicine & Pharmacotherapy, 107, 168–176. 10.1016/j.biopha.2018.07.151 30086463

[mgg3702-bib-0014] Svoronos, A. A. , Engelman, D. M. , & Slack, F. J. (2016). OncomiR or tumor suppressor? The duplicity of microRNAs in cancer. Cancer Research, 76(13), 3666–3670. 10.1158/0008-5472.CAN-16-0359 27325641 PMC4930690

[mgg3702-bib-0015] Xiang, X. , Mei, H. , Qu, H. , Zhao, X. , Li, D. , Song, H. , … Tong, Q. (2015). miRNA‐584‐5p exerts tumor suppressive functions in human neuroblastoma through repressing transcription of matrix metalloproteinase 14. Biochimica et Biophysica Acta, 1852(9), 1743–1754. 10.1016/j.bbadis.2015.06.002 26047679

[mgg3702-bib-0016] Xiong, D.‐D. , Dang, Y.‐W. , Lin, P. , Wen, D.‐Y. , He, R.‐Q. , Luo, D.‐Z. , … Chen, G. (2018). A circRNA‐miRNA‐mRNA network identification for exploring underlying pathogenesis and therapy strategy of hepatocellular carcinoma. Journal of Translational Medicine, 16(1), 220 10.1186/s12967-018-1593-5 30092792 PMC6085698

[mgg3702-bib-0017] Yang, L. , Guo, Y. , Liu, X. , Wang, T. , Tong, X. , Lei, K. , … Xu, Q. (2018). The tumor suppressive miR‐302c‐3p inhibits migration and invasion of hepatocellular carcinoma cells by targeting TRAF4. Journal of Cancer, 9(15), 2693–2701. 10.7150/jca.25569 30087710 PMC6072805

[mgg3702-bib-0018] Yanglin, P. , Lina, Z. , Zhiguo, L. , Na, L. , Haifeng, J. , Guoyun, Z. , … Daiming, F. (2007). KCNE2, a down‐regulated gene identified by in silico analysis, suppressed proliferation of gastric cancer cells. Cancer Letters, 246(1–2), 129–138. 10.1016/j.canlet.2006.02.010 16677757

[mgg3702-bib-0019] Zhou, X. , Wen, W. , Shan, X. , Zhu, W. , Xu, J. , Guo, R. , … Shu, Y. (2017). A six‐microRNA panel in plasma was identified as a potential biomarker for lung adenocarcinoma diagnosis. Oncotarget, 8(4), 6513–6525. 10.18632/oncotarget.14311 28036284 PMC5351649

